# Fragments of the *Fab-3* and *Fab-4* Boundaries of the *Drosophila melanogaster Bithorax* Complex That Include CTCF Sites Are not Effective Insulators

**DOI:** 10.1134/S1607672922010069

**Published:** 2022-03-11

**Authors:** O. V. Kyrchanova, N. Y. Postika, V. V. Sokolov, P. G. Georgiev

**Affiliations:** Institute of Gene Biology, Russian Academy of Sciences, Moscow, Russia

**Keywords:** insulators, dCTCF, regulatory domain, architectural proteins, *Abd-B*, *Fab-7*, *abd-A*

## Abstract

The segment-specific regulatory domains of the *Bithorax* complex (BX-C), which consists of three homeotic genes *Ubx, abd-A* and *Abd-B*, are separated by boundaries that function as insulators. Most of the boundaries contain binding sites for the architectural protein CTCF, which is conserved for higher eukaryotes. As was shown previously, the CTCF sites determine the insulator activity of the boundaries of the *Abd-B* regulatory region. In this study, it was shown that fragments of the *Fab-3* and *Fab-4* boundaries of the *abd-A* regulatory region, containing CTCF binding sites, are not effective insulators.

Chromosome architecture and distant interactions between regulatory elements are one of the key fields of research in modern biology. Today, it became obvious that, to establish correct expression of genes, their regulatory elements specifically interact with each other [1]. Recently, genome-wide 3D studies have shown that chromosomes of higher eukaryotes are organized into topologically associated domains (TADs) [2]. The architectural protein CTCF, which so far is the only comprehensively studied mammalian insulator protein, plays the key role in the organization of TAD boundaries in vertebrates [3].

One of the most convenient models for studying the organization of spatial specific interactions between regulatory elements in vivo is the *Drosophila melanogaster* *Bithorax* complex (BX-C). It consists of three homeotic genes *Ultrabithorax* (*Ubx*), *abdominal-A* (*abd-A*), and *Abdominal-B* (*Abd-B*), which are responsible for the formation of the third thoracic (T3) and all abdominal (A2–A7) segments [4–6]. These genes are regulated by tissue-specific regulatory domains (*abx/bx*, *bxd/pbx*, and *iab-2*–*iab-8*), alternating in the order of the location of the segments that they control (Fig. 1a). Each domain is responsible for the expression of one of the three genes and functions autonomously due to the surrounding insulator boundaries [7, 8]. The regulatory region of the *Abd-B* gene, represented by the *iab-5–iab-8* domains, which are flanked by the *Mcp*, *Fab-6*, *Fab-7*, and *Fab-8* boundaries, has been studied in most detail [4–6] (Fig. 1a). The regulatory elements PRE (Polycomb response element), which recruit the Polycomb complexes, are located near the boundaries [9, 10]. Using *Fab-7* and *Fab-8* as an example, it was shown that the boundaries have two functions: they maintain the autonomy of neighboring *iab*-domains and, at the same time, ensure specific interactions between *iab-* domains and the *Abd-B* promoter [11]. The *Mcp*, *Fab-6*, and *Fab-8*  boundaries contain the binding sites for the homologue of mammalian CTCF protein (dCTCF), and the *Fab-7* and *Mcp* boundaries contain the binding sites for the architectural protein Pita [12, 13].

**Fig. 1.**
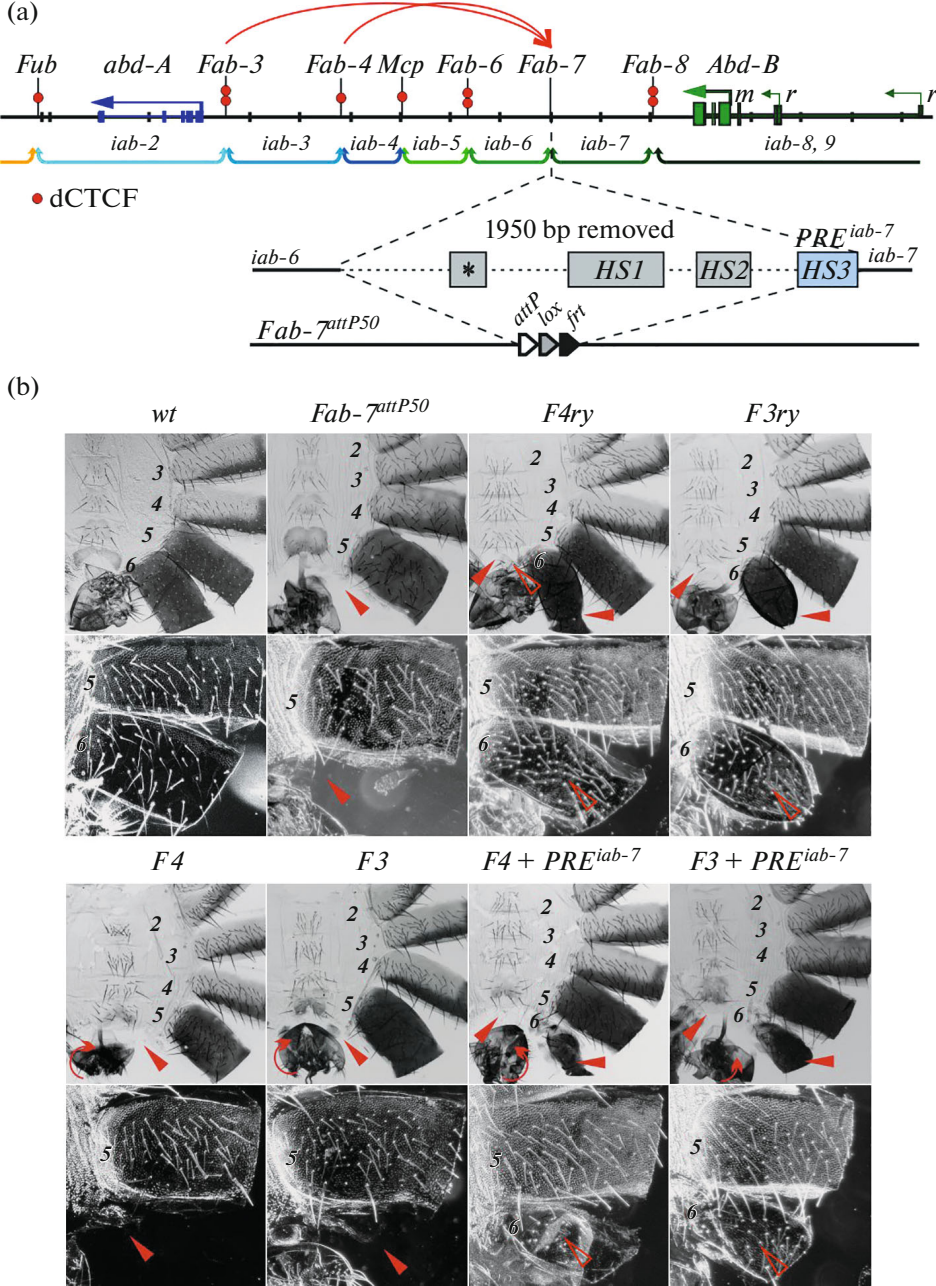
(a) Scheme of the regulatory region of *abd-A* and *Abd-B* genes. Blue and green arrows show the *abd-A* gene transcripts, and blue color show the regulatory region of the *Abd-B* gene, respectively. Curly braces denote *iab*-domains containing segment-specific enhancers (the regulatory region of the *abd-A* gene is shown in blue, and the regulatory region of the *Abd-B* gene is shown in green). Vertical black lines mark the boundaries (*Fub*, *Fab-3*, *Fab-4*, *Mcp*, *Fab-6*, *Fab-7*, and *Fab-8*) of the *iab-2–iab-8,9* regulatory domains, which are responsible for gene regulation and differentiation of segments T3–A8. The dCTCF-binding sites at the boundaries are shown with red circles. Below is a map of the *Fab-7* boundary region that was removed and replaced with *attP*, *lox*, and *frt* sites in the *Fab-7*^*attP50*^ platform. Hypersensitivity sites, HS *, HS1, and HS2, are shown with gray rectangles, and the HS3 site, which is the *PRE*^*iab-7*^ silencer, is shown in blue. (b) Photographs of the male abdomen cuticle in the light and dark field: *wt* (wild type), *Fab-7*^*attP50*^, *F4 ry*, *F3 ry*, *F4*, *F3*, *F4* + *PRE*^*iab-7*^, and *F3* + *PRE*^*iab-7*^. In adult *wt* males, the A7 segment is absent, the A6 sternite is banana-shaped and devoid of bristles, and the A5 sternite is quadrangular and covered with bristles. The A6 tergite has trichomes (dark field) only along the anterior and ventral margins, whereas the entire A5 tergite is almost completely covered with trichomes. In *Fab7*^*attP50*^ males, A6 is absent. The A6 segment of lines *F3 ry* and *F4 ry* is devoid of the sternite (partial transformation into A7) but has a deformed tergite. However, the distribution of trichomes indicates partial transformation into A5. In *F3* and *F4* lines, the complete disappearance of A6 is observed again. In the case of *F4+PRE*^*iab-7*^ and *F3+PRE*^*iab-7*^, the sternite is absent, and the tergite is markedly reduced in size but has extraneous trichomes, which indicates the transformation into A5.

The aim of this study was to attempt to identify the boundaries in the regulatory region of the *abd-A* gene. Previously, regions corresponding to the potential boundaries *Fab-3* and *Fab-4* surrounding the *iab-3* domain, containing two (*Fab-3*) and one (*Fab-4*) binding sites for the dCTCF protein, were found in the regulatory region of the *abd-A* gene [12]. It was shown previously that DNA fragments *Fab-3* (626 bp, *F3*, (3R:16835097..16834472)) and *Fab-4* (784 bp, *F4* (3R:16857582..16856799)) (coordinates are indicated in accordance with Genome Release r6. 41, FlyBase), despite the presence of dCTCF sites, did not exhibit the properties of insulators at the imago stage in the studies of transgenic *Drosophila* lines using model regulatory systems of the *yellow* and *white* genes [14]. Four dCTCF sites also did not block the enhancers of the *yellow* and *white* genes; however, when located in the regulatory region of the *Abd-B* gene instead of the *Fab-7* boundary, they effectively isolate the *iab-6* domain, which ensures the formation of the A6 segment, from the *iab-7* domain, which is responsible for the lysis of the A7 segment in adult male flies [15, 16]. Therefore, it can be expected that fragments *F3* and *F4* can also form a boundary in the BX-C context. To study this issue, we used the previously created *Fab-7*^*attP50*^ platform, in which the *Fab-7* boundary was replaced with *attP* and *frt* sites (Fig. 1a) [17, 18]. The *Fab-7* boundary consists of four hypersensitivity sites for DNAse I: HS * + HS1 + HS2, which form an insulator, and HS3, which is considered a *PRE*^*iab-7*^ silencer [10].

All four sites in the *Fab-7*^*attP50*^ line were removed, which led to premature ectopic activation of the *iab-7* domain by the *iab-6* initiator in all PS11 cells, as a result of which the A6 segment in males was lysed (Fig. 1b), and in females it was transformed into A7. On the basis of the pBluKS plasmid, we created constructs carrying the *attB* site for integration of *F3* or *F4* fragments into the desired place site in the genome at the *attP* site, the *frt* site for subsequent excision of the reporter and plasmid sequences, and the *rosy* (*ry*) reporter gene for selection of positive events. The resulting constructs were injected into the embryos of the *ry*^*–*^ *Fab-7*^*attP50*^ line, which contains a construct on the X chromosome that expresses the φc31 integrase at the preblastoderm stage [18]. The survived flies were crossed with the *yw*;*TM2/MKRS* line carrying mutations in the *ry* gene. Positive events of integration into the platform were selected by the restoration of the color of the eyes by the transgenic *ry* gene to the wild type.

As a result, the lines *F3 ry* and *F4 ry* were created. In both cases, slight restoration of boundary functions was observed, which was manifested as an incomplete formation of the A6 segment (the tergite was deformed, and the sternite either did not develop or consisted of small islets of cells) (Fig. 1b). The distribution pattern of trichomes on the tergite and bristles on the sternite remnants indicated that A6 cells in these lines have the specification characteristic of the A5 segment. This fact indicates that *iab-6* in these tissues is isolated not only from *iab-7* but also from the *Abd-B* gene promoter. However, after deletion of *ry* as a result of Flp-induced recombination between the *frt* sites, a complete lysis of the A6 segment in the *F3* and *F4* lines in males (Fig. 1b) and the transformation of A6 to A7 in females was observed, which suggests a complete loss of insulation. Since the *ry* gene itself does not form the boundary between *iab-6* and *iab-7*, it can be concluded that, in the *F3 ry* and *F4 ry* lines, the *F3* and *F4* fragments cooperate with the *ry* gene in forming the boundary in individual cells of the A6 segment. It is most likely that the *ry* gene promoter is involved in this process. Thus, the studied fragments *F3* and *F4* have a very weak insulator activity. It is important to note that the *F3* and *F4* fragments inserted instead of *Fab-7* caused male and female sterility. In this regard, it can be noted that the reproductive apparatus of homozygous males in both lines is rotated by 30°–180°. In addition, both in *F3* and *F4* lines, a significant decrease in the survival rate of homozygotes was observed. When heterozygous females and males on the *MKRS* or *TM2* balancer were crossed, instead of expected ~33% in progeny no more than 5% of homozygotes survived, and they showed a delay in development (hatched much later than the wild type); when the temperature dropped to 18° C, both *F3* and *F4* homozygotes were not detected at all.

Earlier, it was shown for the *Fab-6* and *Fab-7* boundaries that PREs located in the *iab-6* and *iab-7* domains were involved in the formation of effective boundaries [19, 20]. To investigate the possible involvement of PRE in the activity of the boundaries of the *abd-A* regulatory region, we created constructs in which *PRE*^*iab- 7*^ (227 bp) was added to the *F3* and *F4* fragments (Fig. 1a) and obtained transgenic lines in which *Fab-7* was replaced with *F3 + PRE*^*iab-7*^*ry* and *F4 + PRE*^*iab-7*^*ry*. However, after the excision of *ry* at the *frt* sites, no complete restoration of the boundary between *iab-6* and *iab-7* in the obtained lines was observed. The phenotype of flies of lines *F3 + PRE*^*iab-7*^ and *F4 + PRE*^*iab-7*^ after the excision of *ry* resembled the phenotype of flies of lines *F3 ry*^+^ and *F4 ry*^+^ (Fig. 1b). Thus, *PRE*^*iab-7*^ only partially enhances the weak insulator activity of the *F3* and *F4* fragments in individual cells of the A6 segment.

The obtained results show that the CTCF sites present in the 626-bp fragment of *F3* and 784-bp fragment of *F4* do not form strong insulators, in contrast to the 337-bp fragment of the *Fab-8* boundary, containing two binding sites for the dCTCF protein, which completely blocked the interaction between *iab-6* and *iab-7* [15]. These results are consistent with the conclusions that unknown architectural proteins together with dCTCF are involved in the formation of strong BX-C boundaries [13]. It can be assumed that, in the *iab-3–iab-4* region, strong insulators within the boundaries are not required, which is associated with the peculiarities of regulation in this region. At the same time, the replacement of the *Fab-7* boundary with *Fab-3* or *Fab-4* boundary fragments adversely affects the viability and fertility of flies. This may be due to the ability of these boundaries to form incorrect contacts with their endogenous copies, which disrupts the correct functioning of *abd-A* and *Abd-B* genes. This assumption requires further studies.

## References

[1] Kyrchanova O., Georgiev P. (2021). Mechanisms of enhancer–promoter interactions in higher eukaryotes. Int. J. Mol. Sci.

[2] Kantidze O.L., Razin S.V. (2020). Weak interactions in higher-order chromatin organization. Nucleic Acids Res.

[3] Maksimenko O.G., Fursenko D.V., Belova E.V. (2021). CTCF as an example of DNA-binding transcription factors containing C2H2-type zinc finger clusters. Acta Nat.

[4] Kyrchanova O., Mogila V., Wolle D. (2015). The boundary paradox in the *Bithorax* complex. Mech. Dev.

[5] Maeda R.K., Karch F. (2006). The ABC of the BX-C: the *bithorax* complex explained. Development.

[6] Bender WW (2020). Molecular lessons from the *Drosophila Bithorax* complex. Genetics.

[7] Bowman S.K., Deaton A.M., Domingues H. (2014). H3K27 modifications define segmental regulatory domains in the *Drosophila bithorax* complex. Elife.

[8] Savitsky M., Kim M., Kravchuk O. (2016). Distinct roles of chromatin insulator proteins in control of the *Drosophila Bithorax* complex. Genetics.

[9] Simon, J., Chiang, A., Bender, W., et al., Elements of the *Drosophila* bithorax complex that mediate repression by Polycomb group products, *Dev. Biol*., 1993, vol. 158, no. 1, pp. 131–144. 10.1006/dbio.1993.1174 8101171

[10] Mihaly J., Hogga I., Gausz J. (1997). In situ dissection of the Fab-7 region of the *bithorax* complex into a chromatin domain boundary and a Polycomb-response element. Development.

[11] Kyrchanova, O., Sabirov, M., Mogila, V., et al., Complete reconstitution of bypass and blocking functions in a minimal artificial Fab-7 insulator from *Drosophila bithorax* complex, *Proc. Natl. Acad. Sci. U. S. A*., 2, vol. 116, no. 27, pp. 13462–13467. 10.1073/pnas.1907190116 PMC6613175 31209019

[12] Holohan E.E., Kwong C., Adryan B. (2007). CTCF genomic binding sites in *Drosophila* and the organisation of the *bithorax* complex. PLoS Genet..

[13] Kyrchanova, O., Maksimenko, O., Ibragimov, A., et al., The insulator functions of the *Drosophila* polydactyl C2H2 zinc finger protein CTCF: necessity versus sufficiency, *Sci. Adv*., 2020, vol. 25, no. 6 (13), art. eaaz3152. 10.1126/sciadv.aaz3152 PMC7096168 32232161

[14] Ivlieva T.A., Georgiev P.G., Kyrchanova O.V. (2011). Study of the enhancer-blocking activities of new boundaries in the *bithorax* complex of *Drosophila melanogaster*. Russ. J. Genet.

[15] Kyrchanova O., Mogila V., Wolle D. (2016). Functional dissection of the blocking and bypass activities of the Fab-8 boundary in the *Drosophila bithorax* complex. PLoS Genet..

[16] Postika, NE., Ivlieva, TA., Georgiev, PG., et al., Study of dCTCF insulator activity in *Drosophila melanogaster* model systems, *Doklady Biochem. Biophys*., 2019, vol. 486, no. 1, pp. 187–191. 10.1134/S1607672919030074 31367818

[17] Wolle, D., Cleard, F., Aoki, T., et al., Functional requirements for Fab-7 boundary activity in the *bithorax* complex, *Mol. Cell. Biol*., 2015, vol. 35, no. 21, pp. 3739–3752. 10.1128/MCB.00456-15 PMC4589599 26303531

[18] Bischof J., Maeda R.K., Hediger M. (2007). An optimized transgenesis system for *Drosophila* using germ-line-specific φC31 integrases. Proc. Natl. Acad. Sci. U. S. A..

[19] Kyrchanova O., Kurbidaeva A., Sabirov M. (2018). The *bithorax* complex iab-7 Polycomb response element has a novel role in the functioning of the Fab-7 chromatin boundary. PLoS Genet..

[20] Postika N., Schedl P., Georgiev P. (2021). Mapping of functional elements of the Fab-6 boundary involved in the regulation of the *Abd-B* hox gene in *Drosophila melanogaster*. Sci. Rep.

